# Supplementary material for: Peroxisomal NAD(H) Homeostasis in the Yeast Debaryomyces hansenii Depends on Two Redox Shuttles and the NAD+ Carrier, Pmp47
Source: Biomolecules. 2023 Aug 24;13(9):1294. doi: 10.3390/biom13091294 (PMC10526880; doi:10.3390/biom13091294)
Supplement: Supplementary file 1 [file biomolecules-13-01294-s001.zip › biomolecules-2542382-supplementary.pdf]

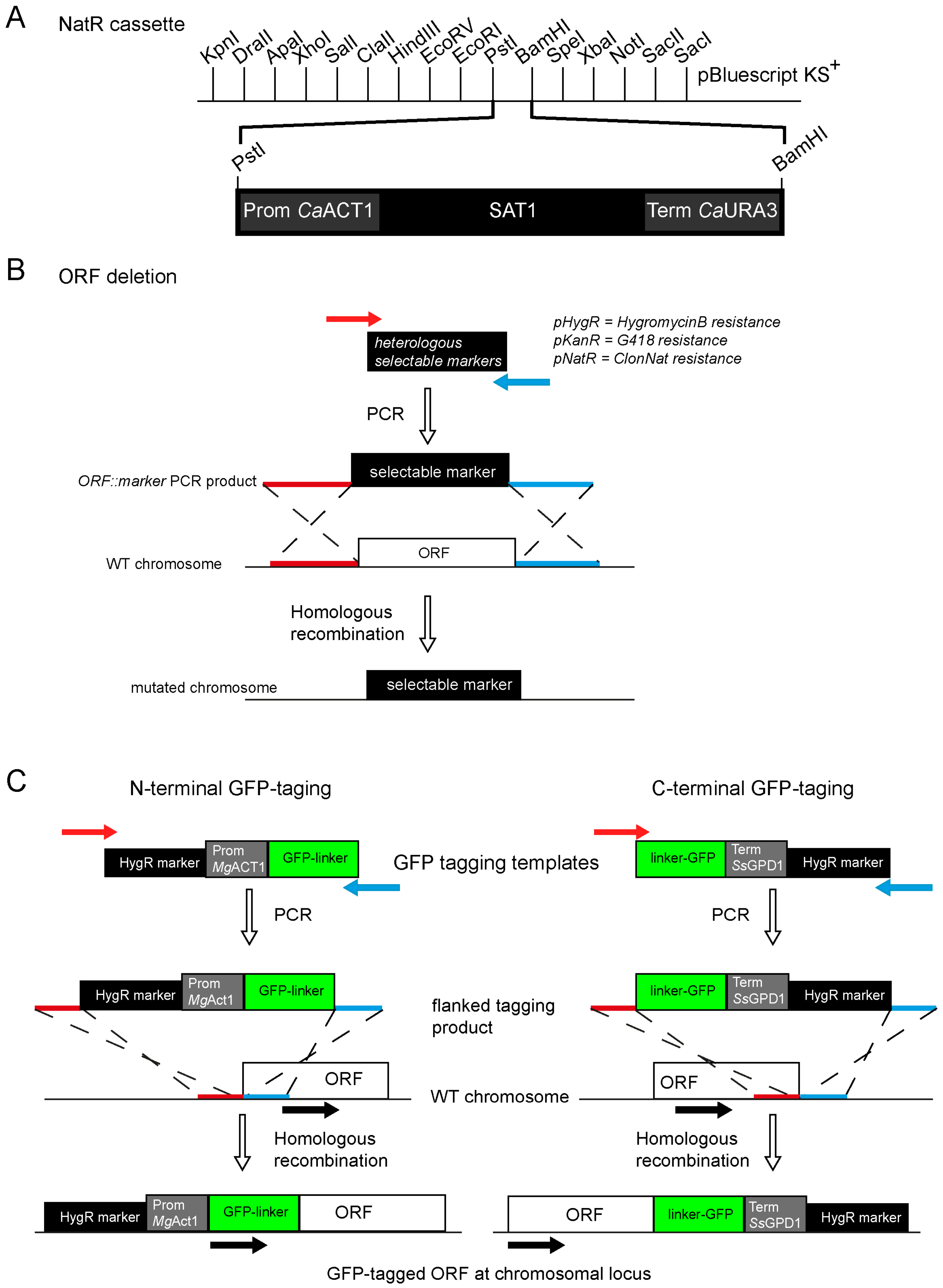
A
NatR cassette
KpnI DraII ApaI XhoI SalI ClaII HindIII EcoRV EcoRI PstI BamHI SpeI XbaI NotI SacII SacI
pBluescript KS+
PstI
BamHI
Prom CaACT1
SAT1
Term CaURA3
B
ORF deletion
heterologous selectable markers
pHygR = HygromycinB resistance
pKanR = G418 resistance
pNatR = ClonNat resistance
PCR
ORF::marker PCR product
selectable marker
WT chromosome
ORF
Homologous recombination
mutated chromosome
selectable marker
C
N-terminal GFP-taging
C-terminal GFP-taging
HygR marker
Prom MgACT1
GFP-linker
GFP tagging templates
linker-GFP
Term SsGPD1
HygR marker
PCR
PCR
HygR marker
Prom MgAct1
GFP-linker
flanked tagging product
linker-GFP
Term SsGPD1
HygR marker
ORF
WT chromosome
ORF
Homologous recombination
Homologous recombination
HygR marker
Prom MgAct1
GFP-linker
ORF
ORF
linker-GFP
Term SsGPD1
HygR marker
GFP-tagged ORF at chromosomal locus

Figure S1. New tools for genome modification of the CTG clade yeast *Debaryomyces hansenii* isolates. (**A**) Diagram depicting the structure of the ClonNat resistance marker adapted from pFA-SAT1 flipper cassette previously used in *Candida albicans* [24,17]. Unique restriction sites in the multiple cloning site of Bluescript KS+ are indicated. Prom *Ca*Act1: *C. albicans ACT1* promoter region. *Ca*URA3 term: *C. albicans URA3* terminator region. SAT1, streptothricin acetyltransferase, which confers resistance to nourseothricin or clonNat. For sequences see Table S5. (**B**) Strategy for deletion of open reading frames using heterologous selectable markers. Three markers were used pHygR, pKanR and pSat1, that confer resistance to HygromycinB, G418 and ClonNat, respectively. These plasmids are flanked by sequences identical to the region directly upstream (red) or downstream (blue) of the target ORF. During initial experiments, large flanking regions of 500 bp to 1kb were cloned into the selection marker plasmids. These large cassettes were subsequently amplified by PCR. However, recently we also developed a PCR-based method in which the selectable marker cassette plasmids can be used as PCR templates using primers that anneal on the cassettes and contain 50 nt 5′ extensions identical to the region directly upstream (red) or downstream (blue) of the target ORF [5]. For primer design see Table S4. Transformation of the PCR products by electroporation results in deletion of the ORF through homologous recombination with high efficiency. (**C**) Two tagging cassettes containing the HygR marker were generated that allow tagging of an ORF in its chromosomal locus resulting in constitutive expression of N-terminally tagged proteins or C-terminal GFP-tagged proteins under control of their own promoter. Prom *Mg*Act1: *Meyerozyma guilliermondii ACT1* gene promoter region. Term *Ss*GPD1: *Scheffersomyces stipitis GPD1* terminator region. See Table S5 for sequences. These plasmids can be used as PCR templates using primers that have 50 nt extensions identical to the region directly upstream of the target insertion site (red) and directly downstream of the insertion site (blue). For primer design see Table S4. Homologous recombination inserts the cassettes into the target site in the genome. Note, for N-terminal tagging, the original translational start codon is replaced by the GFP start codon. For C-terminal tagging, the stop codon of the target ORF is deleted and gly-ala-gly-ala-gly linker followed by GFP is inserted in frame with the ORF.

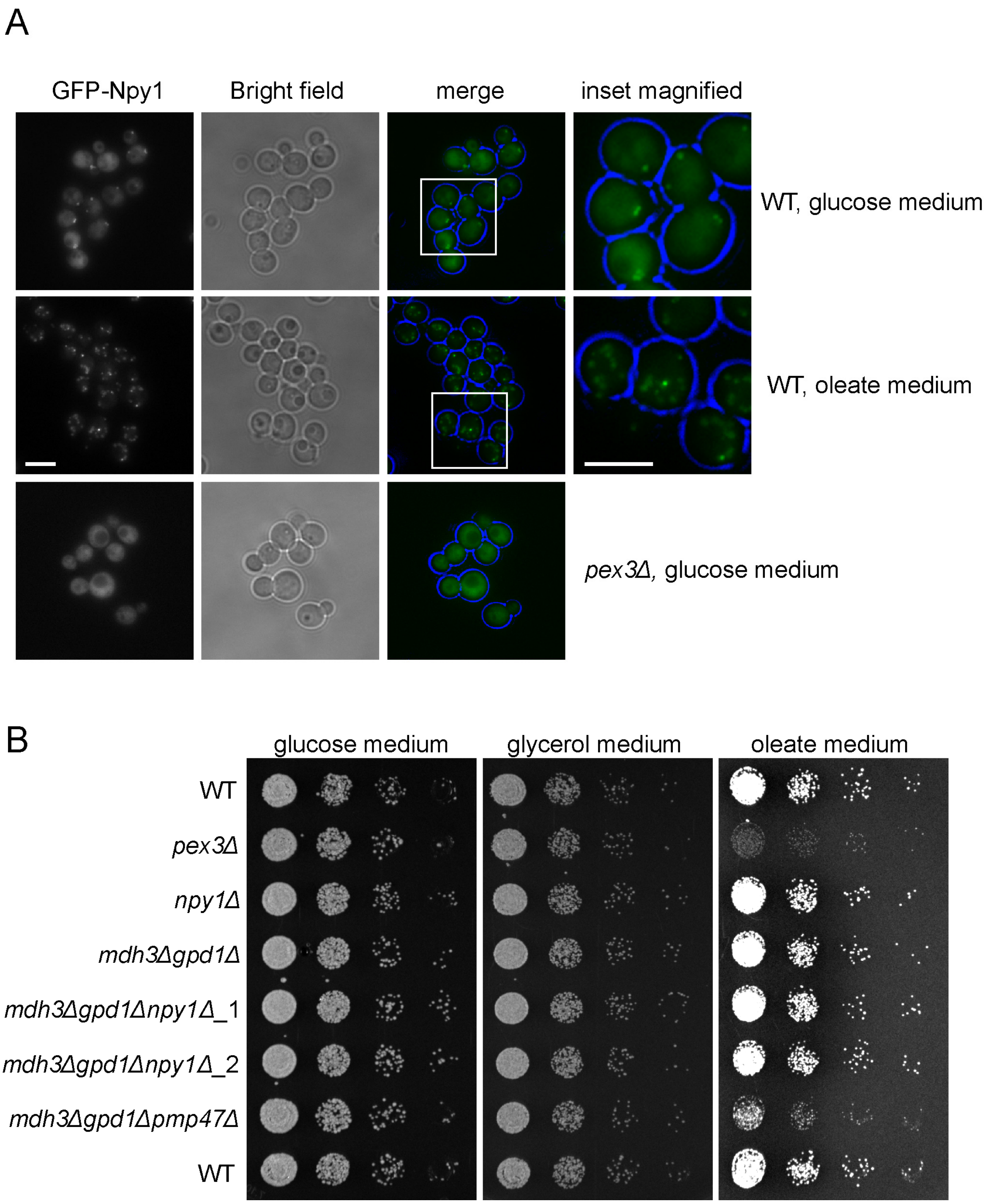

Figure S2. *Dh*Npy1 is a peroxisomal protein not required for growth on oleate. (**A**) Representative epifluorescence microscopy images of wild-type and *pex3Δ D. hansenii* cells expressing mCherry-PTS1 and Npy1 tagged with GFP expressed from its chromosomal locus (GFP-Npy1). Wild-type cells were grown on glucose or oleate media. *pex3Δ* cells were only imaged after growth on glucose medium. Green channel images are merged Z-stacks. Bright field (BF) images were collected in one plane and were processed to highlight the cell circumference in blue in other panels. Scale bar: 5 µm. (**B**) Growth analysis of wild-type and mutant *D. hansenii* cells on media containing glucose, glycerol or oleate as sole carbon source. Two independent *gpd1Δmdh3Δnpy1Δ* strains were analysed.

Table S1. Orthologues of β-oxidation-related proteins identified in *D. hansenii*.

Protein	Genes identified in *D. hansenii*	PTS1/PTS2 prediction	Ortholog in *H. sapiens*	Ortholog in *S. cerevisiae*	Ortholog in *U. maydis*
Acyl-CoA oxidase (Pox1/Fox1)	Q6BVP3, Q6BRD5, Q6BRD8	No	Yes	Yes	Yes
Acyl-CoA Dehydrogenase N (Acad11n)	Q6BX30	PTS1 (-SKL)	Yes	No	Yes
Acyl-CoA Dehydrogenase C (Acad11c)	Q6BQL2	PTS1 (-SKL)	Yes	No	Yes
3-hydroxyacyl-CoA dehydrogenase and enoyl-CoA hydratase (Fox2)	Q6BYL5	PTS1 (-AKI)	Yes	Yes	Yes
3-Ketoacyl-CoA thiolase (Pot1/Fox3)	Q6BVV6, Q6BNX5, Q6BR82, Q6BM30	Q6BVV6: PTS2 (-RLNQVLGHL), Q6BXN5: PTS2 (-RLNQLSGQL), Q6BM30: PTS1 (-SKL), Q6BR82: none	Yes	Yes	Yes
Sterol Carrier Protein 2 (Pox18)-like protein	Q6BYJ2	PTS1 (-AKL)	Yes	No	Yes
Malate Dehydrogenase 3 (Mdh3)	Q6BM17	PTS1 (-SKL)	No	Yes	Yes
Glycerol-3-phosphate Dehydrogenase (Gpd1)	Q6BM03?	Non-consensus PTS2 (-RANQRLQQL)	Yes	Yes	Yes
Carnitine-O-Acetyltransferase	B5RTK8	PTS1 (-AKL)	Yes	Yes	Yes
2,4-Dienoyl-CoA reductase (Sps19)	Q6BVJ4, Q6BH12	PTS1 (Q6BVJ4:-NKL, Q6BH12: -SKL)	Yes	Yes	Yes
Delta3,5-Delta2,4-dienoyl-CoA isomerase (Dci1)	Q6BML0	No	Yes	Yes	Yes
Related to Δ3,Δ2-enoyl-CoA isomerase (Eci1)	Q6BQU9, Q6BZL5	No	Yes	Yes	Yes
Peroxisomal Acyl CoA Thioesterase	Q6BPV5, Q6BZL6, Q6BPV3, Q6BPV4	Q6BPV5: PTS1 (-AKL), Q6BZL6: PTS1 (-PKL), Q6BPV3: PTS1 (-AKL), Q6BPV4: none	Yes	Yes	No
Related to Acyl-CoA Ligase	Q6BWM7, Q6BSB7, Q6BSB6, B5RV06, Q6BWF8, Q6BJ16	Q6BWM7: PTS1 (-SKF), Q6BSB7 & B5RV06: PTS1 (-AKF), Q6BWF8: PTS1 (-SKL). Q6BSB6 & Q6BJ16: none	Yes	Yes	Yes

Very Long Chain acyl-CoA Synthase (Fat1)	Q6BL99	PTS1 (-AKL)	Yes	Yes	Yes
Peroxisomal Half ABC Transporter (Pxa1)	Q6BUD3	No	Yes	Yes	Yes
Peroxisomal Half ABC Transporter (Pxa2)	Q6BWT7	No	Yes	Yes	Yes
Adenine Nucleotide Transporter 1 (Ant1)	Q6BQ51	No	Yes	Yes	Yes
Peroxin 11 (Pex11)	Q6BYZ1	No	Yes	Yes	Yes
Nudix Hydrolase (Npy1)	Q6BV93	PTS1 (-NKL)	Yes	Yes	Yes
Peroxisome Membrane Protein (Pmp47/Ant1/PMP 34)	Q6BI42	No	Yes	Yes	No

Table S2. The list of *D. hansenii* and *S. cerevisiae* strains used in this study.

**Strain Name**	**Genotype**	**Type of Organism**	**Source**
NCYC 102	Wild-type	*D. hansenii*	NCYC
NCYC 3363	Wild-type	*D. hansenii*	NCYC
pex3Δ	NCYC102, *pex3::SAT1*	*D. hansenii*	This study
pex3Δ	NCYC3363, *pex3::SAT1*	*D. hansenii*	This study
mdh3Δ	NCYC3363, *mdh3::hygB^r*	*D. hansenii*	This study
gpd1Δ	NCYC3363, *gpd1::SAT1*	*D. hansenii*	This study
gpd1Δmdh3Δ	NCYC3363, *gpd1::SAT1, mdh3::hygB^r*	*D. hansenii*	This study
pmp47Δ	NCYC3363, *pmp47::hygB^r*	*D. hansenii*	This study
npy1Δ	NCYC3363, *npy1::G418^r*	*D. hansenii*	This study

gpd1Δpmp47Δ	NCYC3363, *gpd1::SAT1, pmp47::G418r*	*D. hansenii*	This study
mdh3Δpmp47Δ	NCYC3363, *mdh3::hygBr, pmp47::G418r*	*D. hansenii*	This study
gpd1Δmdh3Δpmp47Δ	NCYC3363, *gpd1::SAT1, mdh3::hygBr, pmp47::G418r*	*D. hansenii*	This study
gpd1Δmdh3Δnpy1Δ	NCYC3363, *mdh3::hygBr, gpd1::SAT1, npy1::G418r*	*D. hansenii*	This study
fox2Δ	NCYC3363, *fox2::hygBr*	*D. hansenii*	This study
NCYC102 + mCherry-SKL	NCYC102, *ARG1/arg1::SsGpd1pr-yemCherry-SKL (G418r)*	*D. hansenii*	This study
pex3Δ + mCherry-SKL	NCYC102, *pex3::SAT1, ARG1/arg1::SsGpd1pr-yemCherry-SKL (G418r)*	*D. hansenii*	This study
NCYC102 + mCherry-SKL, GFP-*MDH3*	NCYC102, *HygBr MgAct1*pr-GFP-*MDH3, ARG1/arg1::SsGpd1pr-yemCherry-SKL(G418r)*	*D. hansenii*	This study
pex3Δ + mCherry-SKL, GFP-*MDH3*	NCYC102, *pex3::SAT1, HygBr MgAct1*pr-GFP-*MDH3, ARG1/arg1::SsGpd1pr-yemCherry-SKL (G418r)*	*D. hansenii*	This study
NCYC102 + mCherry-SKL, *GPD1*-GFP	NCYC102, *GPD1*-GFP*::HygBr, ARG1/arg1::SsGpd1pr-yemCherry-SKL (G418r)*	*D. hansenii*	This study
pex3Δ + mCherry-SKL, *GPD1*-GFP	NCYC102, *pex3::SAT1, GPD1*-GFP*::HygBr, ARG1/arg1::SsGpd1pr-yemCherry-SKL (G418r)*	*D. hansenii*	This study
NCYC102 + mCherry-SKL, *PMP47*-GFP	NCYC102, *PMP47*-GFP*::HygBr ARG1/arg1::SsGPD1pr-yemCherry-SKL (G418r)*	*D. hansenii*	This study
NCYC102, GFP-*NPY1*	NCYC102, *HygBr -MgAct1*pr-GFP-*NPY1 (HygBr)*	*D. hansenii*	This study
pex3Δ, GFP-*NPY1*	NCYC102, *pex3::SAT1, HygBr-MgAct1*pr-GFP-*NPY1*	*D. hansenii*	This study

BJ1991	*MATα, leu2, trp1, ura3-251, prb1-1122, pep4-3, gal2*	*S. cerevisiae*	[41]
fox1Δ	BJ1991, *fox1::KanMX*	*S. cerevisiae*	Euroscarf
mdh3Δ	BJ1991, *mdh3::LEU2*	*S. cerevisiae*	[11]
gpd1Δ	BJ1991, *gpd1::BLE*	*S. cerevisiae*	[7]
mdh3Δgpd1Δ	BJ1991, *mdh3::LEU2, gpd1::KanMX*	*S. cerevisiae*	[7]
mdh3Δnpy1Δ	BJ1991, *mdh3::LEU2, npy1::BLE*	*S. cerevisiae*	[11]
BJ1991 + *DhPMP47*	BJ1991, + *CTA1pr-DhPMP47* (ARS1/CEN4, *URA3)*	*S. cerevisiae*	This study
mdh3Δ + *DhPMP47*	BJ1991, *mdh3::LEU2, CTA1pr-DhPMP47* (ARS1/CEN4, *URA3)*	*S. cerevisiae*	This study
gpd1Δ + *DhPMP47*	BJ1991, *gpd1::BLE, CTA1pr-DhPMP47* (ARS1/CEN4, *URA3)*	*S. cerevisiae*	This study
mdh3Δ/gpd1 + *DhPMP47*	BJ1991, *mdh3::LEU2, gpd1::KanMX, CTA1pr-DhPMP47* (ARS1/CEN4, *URA3)*	*S. cerevisiae*	This study
mdh3Δnpy1Δ + *DhPMP47*	BJ1991, *mdh3::LEU2; npy1::BLE, CTA1pr-DhPMP47* (ARS1/CEN4, *URA3)*	*S. cerevisiae*	This study
BY4741	*MATa his3Δ1 leu2Δ0 met15Δ0 ura3Δ0*	*S. cerevisiae*	Euroscarf
mdh3Δ	BY4741, *mdh3::SpHIS5*	*S. cerevisiae*	[7]
gpd1Δmdh3Δ	BY4741, *gpd1::KanMX , mdh3::SpHIS5*	*S. cerevisiae*	[7]
BY4741 + *DhPMP47*	BY4741, *CTA1pr-DhPMP47* (ARS1/CEN4, *URA3)*	*S. cerevisiae*	This study

mdh3Δ + DhPMP47	BY4741, *mdh3::SpHIS5, CTA1pr-DhPMP47* (ARS1/CEN4, *URA3)*	*S. cerevisiae*	This study
gpd1Δmdh3Δ + DhPMP47	BY4741, *gpd1::KanMX , mdh3::SpHIS5, CTA1pr-DhPMP47* (ARS1/CEN4, *URA3)*	*S. cerevisiae*	This study
BY4741 + *DhMDH3*	BY4741, *Tpi1pr-DhMDH3* (ARS1/CEN4, *LEU2)*	*S. cerevisiae*	This study
gpd1/mdh3Δ + DhMDH3	BY4741, *gpd1::KanMX , mdh3::SpHIS5, Tpi1pr-DhMDH3* (ARS1/CEN4, *LEU2)*	*S. cerevisiae*	This study
BY4741 + *DhGPD1-GFP*	BY4741, *Tpi1pr-DhGPD1-GFP* (ARS1/CEN4, *URA3)*	*S. cerevisiae*	This study
gpd1/mdh3Δ + DhGPD1-GFP	BY4741, *gpd1::KanMX , mdh3::SpHIS5, Tpi1pr-DhGPD1-GFP* (ARS1/CEN4, *URA3)*	*S. cerevisiae*	This study
BY4741 + *DhPMP47*	BY4741, *Tpi1pr-DhPMP47* (ARS1/CEN4, *LEU2)*	*S. cerevisiae*	This study
gpd1Δmdh3Δ + DhPMP47	BY4741, *gpd1::KanMX, Tpi1pr-DhPMP47* (ARS1/CEN4, *LEU2)*	*S. cerevisiae*	This study
BY4741 + *DhPMP47*-GFP + Pex11-mRFP	BY4741, *Tpi1pr-DhPMP47-GFP* (ARS1/CEN4, *LEU2) + Pex11pr-ScPEX11-mRFP* (ARS1/CEN4, *URA3)*	*S. cerevisiae*	This study
BY4742	MATα his3Δ1 leu2Δ0 lys2Δ0 ura3Δ0	S. cerevisiae	Euroscarf
BY4742 + *HsCPT2^{cyt}*	BY4742, *HsCPT2^{cyt}* (ARS1/CEN4, *URA3)*	*S. cerevisiae*	This study
BY4742 + *HsCPT2^{PTS1}*	BY4742, *HsCPT2^{PTS1}* (ARS1/CEN4, *URA3)*	*S. cerevisiae*	This study
BY4742 + *HsCPT2^{PTS1}* + *DhPMP47*	BY4742, *HsCPT2^{PTS1}* (ARS1/CEN4, *URA3), DhPMP47* (ARS1/CEN4, *LEU2)*	*S. cerevisiae*	This study
mdh3Δ + HsCPT2^{cyt}	BY4742, *mdh3::KanMX, HsCPT2^{cyt}* (ARS1/CEN4, *URA3)*	*S. cerevisiae*	This study

mdh3Δ + HsCPT2^{PTS1}	BY4742, *mdh3::KanMX, HsCPT2^{PTS1}* (ARS1/CEN4, *URA3)*	*S. cerevisiae*	This study
mdh3Δ + HsCPT2^{PTS1} + DhPMP47	BY4742, *mdh3::KanMX, HsCPT2^{PTS1}::URA3*, (ARS1/CEN4, *LEU2)*	*S. cerevisiae*	This study

Table S3. The list of plasmids used in this study.

**Plasmid Name**	**Insert**	**Parental vector**	**Purpose**	**Source**
pHygR	loxP- *S. stipitis TEF1* promoter-CTG adapted HygR ORF-*S. stipitis TEF1* terminator-loxP	pUC19	To generate gene deletions/modifications in *D. hansenii* using HygR as a selection marker	GenScript
pKanR	loxP-S. *stipitis ACT1* promoter-CTG adapted KanR ORF-*S. stipitis ACT1* terminator-loxP	pUC19	To generate gene deletions/modifications in *D. hansenii* using KanR as a selection marker	GenScript
pNatR	*CaACT1* promoter-CTG codon adapted SAT1 ORF-*CaURA3* terminator from plasmid pFA-SAT1	pBlueScript KS(+)	To generate gene deletions/modifications in *D. hansenii* using ClonNat as a selection marker	This study
pSA2	~0.9 kb upstream flank of *DhPEX3 Eco*R1-*Kpn*1 fragment	pNatR	*DhPEX3* deletion cassette containing long flanks of *DhPEX3*	This study
	~1 kb downstream flank of *DhPEX3 Sac*1-*Xba*1 fragment			
pSLV4	~1 kb upstream flank of *DhMDH3 Kpn*1-*BamH*1 fragment	pHygR	*DhMDH3* deletion cassette containing long flanks of *DhMDH3*	This study
	~1 kb downstream flank of *DhMDH3 Xba*1-*Sph*1 fragment			
pSLV19	~1 kb upstream flank of *DhGPD1 Kpn1-Hind3* fragment	pNatR	*DhGPD1* deletion cassette containing long flanks of *DhGPD1*	This study
	~1 kb downstream flank of *DhGPD1 Not*1-*Sal*1 fragment			

pSLV14	~1 kb upstream flank of *DhFOX2* fragment *Kpn*1-*Spe*1 fragment<br>~1 kb downstream flank of *DhFOX2 Sal1-Hind3* fragment	pHygR	*DhFOX2* deletion cassette containing long flanks of *DhFOX2*	This study
pSA4	~1 kb of upstream flank of *DhARG1* *Eco*R1-*Bam*H1 fragment<br>~1 kb of downstream flank of *DhARG1*<br>*Sal*1-*Sph*1 fragment	pKanR	Used as a backbone plasmid for heterologous expression that integrates into the *ARG1* locus. KanR selection marker	[5]
pSA5	~1 kb of upstream flank of *DhARG1* *Eco*R1-*Bam*H1 fragment<br>~1 kb of downstream flank of *DhARG1*<br>*Sal*1-*Sph*1 fragment	pHygR	Used as a backbone plasmid for heterologous expression that integrates into the *ARG1* locus, HygR selection marker	[5]
pSA6	*HygR*, *MgACT1* promoter-CTG codon optimised GFP-PTS1	pSA5	GFP-PTS1 expression for integration into *ARG1* locus.<br>Also used as a PCR template for N-terminal GFP tagging and HygR selection	This study
pSLV35	*MgACT1* promoter-CTG codon adapted yemCherry- PTS1, KanR.	pSA4	mCherry-PTS1 expression cassette for integration into *ARG1* locus	[5]
pSLV37	*SsGPD1* promoter mCherry-PTS1, *SsGPD1* terminator, KanR.	pSLV35	mCherry-PTS1 expression cassette for integration into *ARG1* locus	[5]
pSLV38	GAGAGA linker-CTG codon optimised GFP (with stop codon)-*SsGPD1* terminator	pHygR	C-terminal GFP tagging cassette to be used in *D. hansenii* using HygR marker	This study

pEH116	*TPI1* promoter-MCS-GAGAGA linker-GFP-*PGK1* terminator	ycplac33	C-terminal GFP tagging plasmid with *URA3* marker to be used in *S. cerevisiae.*	Hettema Lab
pEH117	*TPI1* promoter-MCS-GAGAGA linker-GFP-*PGK1* terminator	ycplac111	C-terminal GFP tagging plasmid with *LEU2* marker to be used in *S. cerevisiae.*	Hettema Lab
pEW324	*TPI1* promoter-MCS- *PGK1* terminator	ycplac111	To express untagged *DhPMP47* and *DhMDH3* in *S. cerevisiae.*	Hettema Lab
pES1	*DhGPD1*	pEH116	To express *Dh*Gpd1-GFP in *S. cerevisiae*	This Study
pSLV24	*DhPMP47*	pEH117	To express *Dh*PMP47-GFP in *S. cerevisiae*	This Study
pSLV39	*DhPMP47*	pEW324	To express untagged *DhPMP47* in *S. cerevisiae*	This study
pSLV41	*TPI1* promoter-*DhMDH3-PGK1*-terminator	pEW324	To express untagged *DhMDH3* in *S. cerevisiae*	This study
pEL30	*CTA1* promoter-MCS-*CTA1* terminator	ycplac33	Yeast expression vector with *CTA1* promoter	[42]
pSC120	*CTA1* promoter-*DhPMP47*- *CTA1* terminator	pEL30	To express untagged *DhPMP47* in *S. cerevisiae*	This study
pIJL30	*CTA1* promoter-MCS-*CTA1* terminator	ycplac111	Yeast expression vector with *CTA1* promoter	[43]
pSC132	*CTA1* promoter-*DhPMP47*- *CTA1* terminator	pIJL30	To express untagged *DhPMP47* in *S. cerevisiae*	This study
pAS131	*PEX11* promoter-PEX11-mRFP-PGK1 terminator	ycplac33	*S. cerevisiae* peroxisomal marker	Hettema Lab

pCPT2^{PTS1}	*HsCPT2* (with mitochondrial targeting sequence deleted and C-terminal extension: KLGSGGEAAVKLSQAKSKL), *CTA1* promoter and terminator	ycplac33	CPT2^{PTS1} expression in *S. cerevisiae*	[16]
pCPT2^{cyt}	*HsCPT2* (with mitochondrial targeting sequence deleted), *CTA1* promoter and terminator	ycplac33	CPT2^{cyt} expression in *S. cerevisiae*	[16]

Table S4. The list of primers used in this study.

**Primer Name**	**5′-->3′ sequence**	**Primer Description/Application**
VIP49	GTTTTCCCAGTCACGACG	Used for sequencing, colony PCR and linearization of the gene deletion cassettes.
VIP50	GGAAACAGCTATGACCATG	
VIP3936	CTCGGTACCTGTATTGAAACCACGCGCCAC	To clone 1 kb fragment upstream of *DhMDH3* ORF into the phygR vector.
VIP3937	CATGGATCCTGCTGCTCCGCAAACTGTAAC	
VIP3938	CGCTCTAGACTCTATCGACCAGGGTACTAC	To clone 1 kb fragment downstream of *DhMDH3* ORF into the phygR vector.
VIP3939	CTCGCATGCAATCACCTTGCCTACCCAGTC	
VIP3932	GTGAAACATCAGGGAGAGGC	To confirm cloning/deletion of ~200 bp outside of 1 kb upstream of *DhMDH3* ORF.
VIP3933	TAATCGCTGACAGTGCCATAGC	To confirm cloning/deletion of ~200 bp outside of 1 kb downstream of *DhMDH3* ORF.
VIP3934	TGAACTCGACCGTGCCAATTG	To confirm deletion of *DhMDH3* or the integration of tagging *Dh*MDH3 ORF construct into the genome.
VIP3935	AGCATTAGGACACGCCTTAC	

VIP3940	CACTGGCAAACTGTGATGGAC	To confirm the integration of *HygR* marker into the genome.
VIP3941	GCCATGTAGTGTATTGACCG	
VIP3983	TAGGAACACTGCAAGCGCATC	To confirm the integration of *KanR* marker into the genome.
VIP3984	AACAGCGATCGCGTATTTCG	
VIP4112	AACACATACATAAACGAGCTCAAAATGTCACAATATAGAGCCAATC	To clone *DhGPD1* into pEH116 (plasmid used for expression in *S. cerevisiae* cells).
VIP4113	CAGGTCGACTCTAGAGGATCCTTTGAATAATGAATGGTCTCC	
VIP4089	CATGGGTACCACTATCCCCACTGGCACTTG	To clone 1 kb fragment upstream of *DhFOX2* ORF into the phygR vector.
VIP4090	CATGACTAGTTGTTAAGTTCCTTGCCGCTC	
VIP4127	GATCGTCGACAGGCTAAGATCTAAGCTAGC	To clone 1 kb fragment downstream of *DhFOX2* ORF into the phygR vector.
VIP4100	CTAGAAGCTTGTGGCCACCAGAAGTCTTTC	
VIP4093	TTCTAACCTGTCCCATCAAG	To confirm cloning/deletion of ~200 bp outside of 1 kb upstream of *DhFOX2* ORF.
VIP4094	CAACAATAACATCCCATGCCG	To confirm cloning/deletion of ~200 bp outside of 1 kb downstream of *DhFOX2* ORF.
VIP4095	ATCAGCAACGGCAATTCCAC	To confirm deletion of *DhFOX2*.
VIP4096	ATGGTATGTCTGCTAAGGTC	
VIP81	GTTTGTATTCTTTTCTTGC	Anneals to the *TPI1* promoter in pEH116, used for sequencing.

VIP272	CCCATTAACATCACCATC	Reverse primers within *GFP* ORF, used to confirm expression and sequencing.
VIP466	TTGTCGGCCATGATGTATACG	
VIP4162	CATGGGTACCCATCGATGCCAATACAACCG	To clone 1 kb fragment upstream of *DhGPD1* ORF into the pNatR vector.
VIP4163	CATGAAGCTTGGCTCTATATTGTGACATTGG	
VIP4164	CATGGCGGCCGCAGATCAGCAACGTTAAGCCG	To clone 1 kb fragment downstream of *DhGPD1* ORF into the pNatR vector. VIP4165 was also used to confirm the integration of *GPD1* tag into the genome.
VIP4165	CATGGAAGCTCATCCAGATCACCGGATAGAG	
VIP4166	AACTCGCAACTGGACAAGAG	To confirm cloning/deletion of ~200 bp outside of 1 kb upstream of *DhGPD1* ORF.
VIP4167	GGCCAAAGGTTACACGTAAC	To confirm cloning/deletion of ~200 bp outside of 1 kb downstream of *DhGPD1* ORF.
VIP4168	CGAAATTGCTCTGGTGGTTG	To confirm deletion of *DhGPD1* (VIP4169 was also used to check the integration of *GPD1* into the genome).
VIP4169	GGTGACAATGCTAAATCGGC	
VIP3397	AGCACACACCCACAACAAC	Within the *NatR* ORF. To check the integration of *SAT1* marker into the genome.
VIP3901	AGACAGCTCCTTGGCATACG	
VIP4257	AACACATACATAAACGAGCTCAAAATGGCCGAAA TTGAAGAACTTGCCC	To clone *DhPMP47* ORF into pEH117 (plasmid used for expression in *S. cerevisiae* cells).
VIP4238	CCTTTACTCATTGCACCCGCCCCTGCTCCCTGCAGT TTAACAGCATTCTTCTTC	

VIP4398	GTGTTGAAGAATTGACTAATAGGTCAGAAAAGGT AGTAACAAAGAGTAACAAACAACAAAACGGGGA TCCATGCATACTAG	To generate *DhPMP47* deletion construct using either *hygR* or *KanR* markers in pHygR or pKanR, respectively.
VIP4462	CGTCAACATTTTAAAATGGCTTGATAATATATTGA AGTATTTAACCAAATGCATACTTATATACTCCTGC AGGTCG ACTCTAGAG	
VIP4460	CGATAAGACTGCAAGTGTCGTGTATATAAATTGCG TCGGTATAGCTGACAAAATCAGATAATGAAGAAA TCGGGGATCCATGCATACTAG	To generate *DhNPY1* deletion construct using either *hygR* or *KanR* markers in pHygR or pKanR respectively.
VIP4461	ATACACTTTATAGTCTATAGAATAAAATTTAAGTA TTTTCCGATTCAATTCTAGAAATGTAACAGCCATC CTGCAGGTCGACTCTAGAG	
VIP4425	ACGTTACAGACTCGTTCTGC	Outside of the flanks used for deleting *DhPMP47* ORF. To confirm *DhPMP47* deletion (VIP4426 was also used to confirm the integration of *PMP47* tag into the genome).
VIP4426	CTATGCGGATGTTTATGCGG	
VIP4427	CTTGTTCGCAGGTGTGTTAC	Within the *DhPMP47* ORF. To confirm *DhPMP47* deletion (VIP4427 was also used to confirm the integration of *PMP47* tag into the genome).
VIP4428	GACAATCGCTTTGAACGTAG	
VIP4513	AACAGCTTCCAGCATGCTTC	To confirm *DhNPY1* deletion (VIP4513 was also used to confirm the integration of *NPY1* tag into the genome).
VIP4514	CTCTATGTCCGCATATGAGG	
VIP4515	CTGGGTGTGGTTCTAGAGTC	To confirm *DhNPY1* deletion. (VIP4516 was also used to confirm the integration of *NPY1* tag into the genome).
VIP4516	TTACCACTGCTCCAGTCTTC	
VIP4517	ACGGATCGAATTCGTGGAAATCTATCATTAGTAGC CAGTTATCAATCTAATAAGTCAAGACGAAGTTATG GAATGATCCAGAGG	To tag *DhMDH3* in *D.hansenii* genome.
VIP4519	TAATAACGACAATGGTTGCCCAATGCCTCCTGCTG CTCCGCAAACTGTAACTTTAACCATTGCGCCAGCT CCTGCACCTTTGTATAGTTCATCCATGCC	

VIP4559	CGAGGCCCATCTTCACATGTGACTCAAAGTCATATAACCATGATGGGGTACTAAATGTTACTTAAACGGATCGAATTCGTGGAAATC	To extend the homology arms of *DhMDH3* tagging construct.
VIP4560	GCAACCCCATTTGCATTAACCACATCAAATAACGACAATTCGCTCACTTGCGGGTTTAACTTTAATAATAACGACAATGGTTGCCC	
VIP465	CCACACAATCTGCCCTTTCG	To confirm the integration of GFP tags in *D. hansenii*.
VIP467	CCATGTGTAATCCCAGCAGC	
VIP4410	CATCTGCAGCTATAATTTCGAACCAGTTACG	Complementary to the sequence near the end of *DhMDH3* ORF. To confirm the integration of *MDH3* tag in *D. hansenii* genome.
VIP4664	TTTGGAATGGGGCCCGGCCATTAGCCCGATAAGACTGCAAGTGTCGTGTATATAAATTGCGTCGGTATAGCTGACAAAATCAGATAATGAAGAAATCGAAGTTATGGAATGATCCAGAGG	To tag *DhNPY1* in *D.hansenii* genome.
VIP4665	ATAATAACTATCTTGTTTCAGCTTACTGATTGATGATTGAAATCGAATCGAAAGTCTTGGTATCTTACTGAAAACGCCCATTGCGCCAGCTCCTGCACCTTTGTATAGTTCATCCATGCC	
VIP4704	ACGTATCCATCTCATCTTAGATAAGTTCATCTTCGTATCAGAAAGGCGTAAGTGTAGTATTATTGAAAGATTTGGAATGGGGCCCGGCCATTAG	To extend the homology arms of *DhNPY1* tagging construct.
VIP4705	AACTATCTCTGCTCCAAAGTATGAGCTATGATCTTGTCCATGAATAGGATTTAATACGTTACCCGACATCTTATAATAACTATCTTGTTTCAGC	
VIP4751	CTGGCTCCGTCAAATTGGTCCAAATCTTCAAATTAATGTGAAAATGAAGAAGAATGCTGTTAAAGGATCCGGTGCAGGAGCTGGCGCAGTCGACCTCGAGATG	To tag *DhPMP47* in *D.hansenii* genome.
VIP4752	CTCTCGGCAACTGACGTCAACATTTTAAAATGGCTTGATAATATATTGAAGTATTTAACCAAATGCATACTTATATACTCTACCCAATCTATCTTCTGAGGTG	
VIP4753	AATTGATTCAATCGATCACTACAGCCGCGTTTTTATTCTACTTTAAAGAGGAATTATTAACTGGCTCCGTCAAATTGGTC	To extend the homology arms of *DhPMP47* tagging construct.

VIP4754	CTCATTTATTGGGCAGAAAAATGAAGCATAAATCCAGCTACTGCGCATATGTTGCAGTATCACCAAATTTTTCGGGCTCTCGGCAACTGACGTCAAC	
VIP4780	CATGCGGCCGCACCCGCTCTTGACGGTTAC	To clone *MgACT1* promoter-CTG codon optimized *mCherry* into pSA5. VIP4781 introduces PTS1 signal (PLH-SKL) to the end of *mCherry,* thus creating for peroxisomal marker *mCherry-PTS1*.
VIP4781	CAGGTCGACCTAGAGTTTTGAGTGCAGTGGTTTATATAATTCATCCATACCACC	
VIP4798	CATGCGGCCGCCGAAGTTATATCTGATGTCTC	To amplify *PsGPD1* promoter and clone it into pSLV35 (for replacing with *MgACT1* promoter).
VIP4799	CAGCTGCAGGATTGATTATGACTATAATGTGTG	
VIP3967	CAATGAATTCCCTGTAGTTGTAGATGCCAC	To clone 916 bp fragment upstream of *DhARG1* ORF into pKanR as *Eco*RI-*Bam*HI fragment
VIP3968	CAATGGATCCGGCAATAGTGATCGGATTG	
VIP3969	CAATGTCGACTAATCAGCAGTCCAGTACTC	To clone 947 bp fragment downstream of *DhARG1* ORF into pHygR or pKanR as *Sal*I-*Sph*I fragment
VIP3970	CAATGCATGCATGGGGACAAGTTGGCTAGATG	
VIP4016	AGGAGCGCGGTATATAGATC	Sequence outside of ~1 kb flanks of *DhARG1*. To confirm the integration of mCherry into the *ARG1* locus.
VIP4019	CAGCGGGTATAGTTGGAATG	
VIP4793	GAAGATGGTGGTGTTGTTAC	To confirm the integration of mCherry into *DhARG1* locus.
VIP4877	TCCCATTATTTGAAGCTACTTATCAAATTATATACGGTGATGAATCTATTCAAAACTTGCCAAACTTATTAGAAGACCATTCATTATTCAAAGAATTCGAGCTCGGTACCCG	To tag *DhGPD1* in *D. hansenii* genome.
VIP4878	CATGCTACTGGTTGTCTAACCAAAAAAAAAAGGCGTCAAAATGAAACGCATCTAATATACATGAAACGGCTTAACGTTGCTGATCTACCTGCAGGTCGACTCTAGAG	

VIP4879	AGAAGCAGAAAAGAAATTATTGAATGGCCAATCCTCGCAAGGTATCATCACTGCAAAGGAAGTCCATGAGTTATTAAGCAATGTTGGTAAGACTGATCAATTCCCATTATTTGAAGCTAC	To extend the homology arms of *DhGPD1* tagging construct.
VIP4880	TCGTATGTATTATAGTAATAATAAAAATCAATGATATTGTAATATTCTGTATATTTTCTATGTGATAAATAAATAACGACAAACTTAAAAATAAATTTGTCATGCTACTGGTTGTCTAAC	
VIP3286	TGAAGCTTCGTACGCTGCAG	To amplify CaACT1 promoter-CTG codon adapted SAT1 ORF-CaURA3 terminator from pFASAT1 and clone it into pBLUESCRIPT KS(+)
VIP3287	GCTGGATCCATGCAGGACCACCTTTGATTG	
VIP3856	CACTGGTACCTGAAGCACTCGAGTTGAAG	To clone ~1 kb fragment upstream of *DhPEX3* ORF into pNatR
VIP3857	CAATGAATTCGCATGCTTAGTAGTTTTGCTTG	
VIP3858	CAGTTCTAGACTTAATGACCTCTTCACATCG	To clone ~1 kb fragment downstream of *DhPEX3* ORF into pNatR
VIP3859	CAATGAGCTCTTCCTACTACCAGACCTACC	
VIP3872	CGCTGAAGCTGATGTAGATC	To confirm the *PEX3* deletion.
VIP3873	GTGATTAATCCTGGCGACTC	
VIP3901	AGACAGCTCCTTGGCATACG	Within the *SAT1* ORF. To confirm the integration of SAT1 marker into the genome.
VIP3397	TAGCACACACCCACAACAAC	
VIP5180	CATGCTGCAGCTATAATTTCGAACCAGTTACG	To clone *DhMDH3* ORF into pEW324 plasmid.
VIP5182	CATGGAGCTCAAAATGGTTAAAGTTACAGTTTGCGG	

VIP5183	GACCCGATACTTTCTTCGCAACTGATGCTGTAGCA TGTACCAAAG	To change the CTG codon present in *DhMDH3* ORF.
VIP5184	CTTTGGTACATGCTACAGCATCAGTTGCGAAGAAA GTATCGGGTC	
PMP47f	GGGGGAGCTCATGGCCGAAATTGAAGAACT3	To express untagged *Dh*PMP47 in *S. cerevisiae*, the PMP47 ORF was PCR amplified from pSLV24 plasmid with the oligonucleotides and subcloned using Sac1 and XbaI restriction sites in pEL30 or pIJL30 vector downstream of the oleate-inducible CTA1 promoter.
PMP47r	GGGGTCTAGATTATTTAACAGCATTCTTCTTCA	

Table S5. The sequences of promoters, terminators and antibiotic resistance ORFs used in this study.

Name	DNA sequence
S. stipitis TEF1 promoter region	GGAATGATCCAGAGGCGCGACATTTATGCAGACAATTTGTGTTTTGTCGCAAACGATGTTATAGC GAAATTTTTCACTCTGTCAGATAAATGGATTTTGTCAAAAGGGGGAAGTAGAAGGAGAATGGGC CCGAGATGTTCTGCCAAATTCTCAGTAGCATAATGTGAAAGAAGCCCTTACATTGTCCAGCCTCT GGCATCATTAAAAACCGTAGCGGAAACCAATTGTCTCTGTTCTTCCCTGGCACACCCTGGTAGCC CCATCCAGTTGTAGTACATCTCACACGCTGGCAACTTGGGACAATCAGCAACTTTTTTTTCTTTTA ATTTTTTCAGCGCGACATTTTGCCTCTTCTGCGAGAACAGACTTTTTCACCTCCATCTCACCCCCCT TTGCACTTATATAAATTGGACCAGTTCCTCCCATTGTAGAAAAAATTTTGCTGGACCTTTTTCTCTT TTTTTTGTCCTTTAGTTTCATACAATCTAAGTCTATCTACA
S. stipitis TEF1 terminator region	GCTGATTAATTTACGTATATTCAGTTTAATATCAATACGTTAGCTACATTTCCAATGAACGATACT AGATATTGTTTAGGATTATTGAACTGGTATAGATAATTTTAGTGTATATTCATGTACTTGATAAAT GTAATAATATGTGAAAATGTAGTTGTACATTAACTGATAGACAACATGCTGGAGTATATGGCATT AAGGTTGCTACAAAGTAGAAGCAACCTAGACACACCTCAGAAGATAGATTGGG
S. stipitis ACT1 promoter region	AAGTTCCGAGCTTCAGCAAACGCTTGTGTGGAAAGCTCCACCAGTGCTAAGGTGGAGTCGGGTTG GGGAAATGTCGCGAACGACACAATTTTTCAGCTCAGACGGCACCCCACCAAAAGAATGATAGCA GATAGCCTGGAGAGAGCCCAGATCAGCCAAAGAATAGCACTAATATACAAATAATACGAAACC CCAAAATACGACATTGTCCTCCCTTATACACACAGATGTGGGCTATTTGTGGATGCCAAAATATA CCCAATCATGTCGCTATCTAGTGTCTTTTGACTTATCTTCCACATTGTTCCCTCTGTGTAGCATGAG CACTCAGCAATGTCGCGTGTCGTGCAAATTTTTCCTTGTGTGCGACTTTCCCACCCACCGATATTT ATAACCAACGCAGTTTTTCTTTTCGTGAGCACAATCCCCTTTTCTTCTTTTTTCAGTAGGTTTCTGTA ATATTAGTACAATCCCTTATATTATAATCATATAGATCAAAC
S. stipitis ACT1 terminator region	AACCACTTGCAAAATCCTTTGTATTCTTGTTCTGCAAACATTTTGTCCAATCTCTTATCTTTCTACG ATGTTGAAGAACATACATTTTTGTTAGTCCAGCTTGTTATCCTTTTTTTATTAAATAAAAAATTTTA TTGTTAAAGTTGTTTTTCTTCTTTGTCTGTCCTGTTTTAACTCATATATATTCTATATATTACAGAAA GAAAGTAGATCATCTAAAATAAACATTTATTTGATGAGACAAAGTAGGTTTTTTAGAATACAATG TAGCATCCTCTAATATACAATAGAGTTGTTTTATAAAAGCGATACATTTAATATTATGACAGGACT GGTGAAGTGTATAGAATTGTGCTAATCTGGCAAACGAACCCAAGAAGACGTTAAAGCAAATAGT GCA

S. stipitis GPD1 promoter region	ATCTGATGTCTCAAATAAGGTATCAAGACAACGAATCAATGATCAACCAAGAAAGATTATTAGA<br>GAAATTGGATGGATCAGCTGTGCTTGTGGAAACACTGATACGACATGCCAGTAAACGAAGTAGA<br>AAAAACTAACTGTATATTCGCTAACAAAAAAATTGTATTATTTTTAGACATCGTAAATGGGGCTG<br>TAGCCCTAATTATTTTTCATTTCTCGTGATCTGCACGTGTACTGTTGATTTTTTTGTGCGATTGAAAT<br>TATATTGTGCTGTTATCTTAATAAAATCAGTCATACCTTTTTTTTTTTGGGTTTTTGTTGTTTAATTTG<br>TGATAAACATCCCATGAGGAACAGCGAGAAAGTTTTTTGCTGTTCACTTTCTCGTTCAACTTTTGC<br>AAAGTAAAGTAAAGAAAAAAAAAATTTCCCTCGTCATCTTGATTTTTACTTCTTCTTCTTCTTCTTC<br>TTTTTCCTTTCTTCACACATTATAGTCATAATCAATC
CTG-adapted hygR	ATGGGTAAAAAGCCTGAACTCACCGCGACGTCTGTCGAGAAGTTTCTAATCGAAAAGTTCGACA<br>GCGTCTCCGACCTAATGCAGCTCTCGGAGGGCGAAGAATCTCGTGCTTTCAGCTTCGATGTAGGA<br>GGGCGTGGATATGTCCTACGGGTAAATAGCTGCGCCGATGGTTTCTACAAAGATCGTTATGTTTAT<br>CGGCACTTTGCATCGGCCGCGCTCCCGATTCCGGAAGTGCTTGACATTGGGGAATTTAGCGAGAG<br>CCTAACCTATTGCATCTCCCGCCGTGCACAGGGTGTCACGTTGCAAGACCTACCTGAAACCGAAC<br>TACCCGCTGTTCTACAGCCGGTCGCGGAGGCCATGGATGCGATCGCTGCGGCCGATCTTAGCCAG<br>ACGAGCGGGTTCGGCCCATTCGGACCGCAAGGAATCGGTCAATACACTACATGGCGTGATTTCAT<br>ATGCGCGATTGCTGATCCCCATGTGTATCACTGGCAAACTGTGATGGACGACACCGTCAGTGCGT<br>CCGTCGCGCAGGCTCTCGATGAGCTAATGCTTTGGGCCGAGGACTGCCCCGAAGTCCGGCACCTC<br>GTGCACGCGGATTTCGGCTCCAACAATGTCCTAACGGACAATGGCCGCATAACAGCGGTCATTGA<br>CTGGAGCGAGGCGATGTTCGGGGATTCCCAATACGAGGTCGCCAACATCTTCTTCTGGAGGCCGT<br>GGTTGGCTTGTATGGAGCAGCAGACGCGCTACTTCGAGCGGAGGCATCCGGAGCTTGCAGGATC<br>GCCGCGGCTCCGGGCGTATATGCTCCGCATTGGTCTTGACCAACTCTATCAGAGCTTGGTTGACG<br>GCAATTTCGATGATGCAGCTTGGGCGCAGGGTCGATGCGACGCAATCGTCCGATCCGGAGCCGG<br>GACTGTCGGGCGTACACAAATCGCCCGCAGAAGCGCGGCCGTCTGGACCGATGGCTGTGTAGAA<br>GTACTCGCCGATAGTGGAAACCGACGCCCCAGCACTCGTCCGAGGGCAAAGGAATAA
CTG-adapted KanR	ATGGGTAAGGAAAAGACTCACGTTTCGAGGCCGCGATTAAATTCCAACATGGATGCTGATTTATA<br>TGGGTATAAATGGGCTCGCGATAATGTCGGGCAATCAGGTGCGACAATCTATCGATTGTATGGGA<br>AGCCCGATGCGCCAGAGTTGTTTCTAAAACATGGCAAAGGTAGCGTTGCCAATGATGTTACAGAT<br>GAGATGGTCAGACTAAACTGGCTAACGGAATTTATGCCTCTTCCGACCATCAAGCATTTTATCCGT<br>ACTCCTGATGATGCATGGTTACTCACCACTGCGATCCCCGGCAAAACAGCATTCCAGGTATTAGA<br>AGAATATCCTGATTCAGGTGAAAATATTGTTGATGCGCTTGCAGTGTTCCTACGCCGGTTGCATTC<br>GATTCCTGTTTGTAATTGTCCTTTTAACAGCGATCGCGTATTTCGTCTCGCTCAGGCGCAATCACG<br>AATGAATAACGGTTTGGTTGATGCGAGTGATTTTGATGACGAGCGTAATGGCTGGCCTGTTGAAC<br>AAGTCTGGAAAGAAATGCATAAGCTATTGCCATTCTCACCGGATTCAGTCGTCACTCATGGTGAT<br>TTCTCACTTGATAACCTTATTTTTGACGAGGGGAAATTAATAGGTTGTATTGATGTTGGACGAGTC<br>GGAATCGCAGACCGATACCAGGATCTTGCCATCCTATGGAACTGCCTCGGTGAGTTTTCTCCTTCA<br>TTACAGAAACGGCTTTTTCAAAAATATGGTATTGATAATCCTGATATGAATAAATTGCAGTTTCAT<br>TTGATGCTCGATGAGTTTTTCTAA
CTG codon-adapted mCherry-PTS1	ATGGTTTCAAAAGGTGAAGAAGATAATATGGCTATTATTAAAGAATTTATGAGATTTAAAGTTCA<br>TATGGAAGGTTCAGTTAATGGTCATGAATTTGAAATTGAAGGTGAAGGTGAAGGTAGACCATATG<br>AAGGTACTCAAACTGCTAAATTGAAAGTTACTAAAGGTGGTCCATTACCATTTGCTTGGGATATTC<br>TGTCACCACAATTTATGTATGGTTCAAAAGCTTATGTTAAACATCCAGCTGATATTCCAGATTATT<br>TAAAATTGTCATTTCCAGAAGGTTTTAAATGGGAAAGAGTTATGAATTTTGAAGATGGTGGTGTTG<br>TTACTGTTACTCAAGATTCATCATTACAAGATGGTGAATTTATTTATAAAGTTAAATTGAGAGGTA<br>CTAATTTTCCATCAGATGGTCCAGTTATGCAAAAAAAAACTATGGGTTGGGAAGCTTCATCAGAA<br>AGAATGTATCCAGAAGATGGTGCTTTAAAAGGTGAAATTAAACAAAGATTGAAATTAAAAGATG<br>GTGGTCATTATGATGCTGAAGTTAAAACTACTTATAAAGCTAAAAAACCAGTTCAATTACCAGGT<br>GCTTATAATGTTAATATTAAATTGGATATTACTTCACATAATGAAGATTATACTATTGTTGAACAA<br>TATGAAAGAGCTGAAGGTAGACATTCAACTGGTGGTATGGATGAATTATATAAACCACTGCACTC<br>AAAACTCTAG

CTG codon-adapted GFP	ATGAGTAAAGGAGAAGAACTTTTCACTGGAGTTGTCCCAATTCTTGTTGAATTAGATGGTGATGTT<br>AATGGGCACAAATTTTCTGTCAGTGGAGAGGGTGAAGGTGATGCAACATACGGAAAACTTACCC<br>TTAAATTTATTTGCACTACTGGAAAACTACCTGTTCCATGGCCAACACTTGTCACTACTTTCACTTA<br>TGGTGTTCAATGCTTTTCAAGATACCCAGATCATATGAAACGGCATGACTTTTTCAAGAGTGCCAT<br>GCCCGAAGGTTATGTACAGGAAAGAACTATATTTTTCAAAGATGACGGGAACTACAAGACACGT<br>GCTGAAGTCAAGTTTGAAGGTGATACCCTTGTTAATAGAATCGAGTTAAAAGGTATTGATTTTAA<br>AGAAGATGGAAACATTCTTGGACACAAATTGGAATACAACTATAACTCACACAATGTATACATC<br>ATGGCAGACAAACAAAAGAATGGAATCAAAGTTAACTTCAAAATTAGACACAACATTGAAGAT<br>GGAAGCGTTCAACTAGCAGACCATTATC
M. guilliermondii ACT1 promoter region	ACCCGCTCTTGACGGTTACCCAATGCGGTTATAAGCCAACAGTCTGTTGTGCGACTAGGCTCGCTT<br>GGCACCTGCACAGATGCTGCGACAGCTCTCACGCACAGAAATGGTCACCTAGAGTCGATTTCCGC<br>GCCTCGTTGCCGCCGGTCTCCGCGCGGTGAATCCTGTACATAGTCATCTCCGATTCACTTTCACTA<br>GACGAATCCGGCACATGAGTGATCCGGCGTGCACACAATAGCAATCTCCCTGCACACACCGGGA<br>CGCGATTGCCGGGTAATCCCTGGTTGGGTCGTTTCTGCCTCGTTGTTTGATACCAGCGCTCACCCC<br>TTTCGAAAAATTTACTTTTGACTAGGTATTAATATAGTATAGCAAA
C. albicans ACT1 promoter region	GTCGAGCGTCAAAACTAGAGAATAATAAAGAAAACGATCTTTTCAAAAAGAAAAAACCTTTTAG<br>TTTTCCTTTGTTGTTGTTGTGGGTGTGTGCTATTTATATTATATAGTTTACTCATAATACCATAAAAT<br>ATTCGGTTTGATTAGGTTATTTTAATAAGCTAATTTGTTTCTAATCGTGTAATTTATGCTGTGTATAT<br>TAAGTAGTGTGTGCACTGCCCAAAAATGTTTGTTGTTTATAGTCGGTTAAAGAGAAAAAAGAAAA<br>AAAGATCCATACACACACGTTAATTAGTTGTTCAACGTAATACACTCATATTTTGTTCTTATTTGCT<br>TTCGGTCGCTGTTCTCACCAAGATTTATTGCCAACGAAACAATTTTTTTTTATATATTTTCAGATTTT<br>TCTTTTTTTCCTTTCCTTTCCTTTTCTAATTTTCACTCCTGGTTTTCTTTCTTTCTTAGAAACATTATCT<br>CGATATTAATATTAAAAAAATATAATCATTCAAA
CTG codon-adapted SAT1	ATGGACGGTGGTATGTTTTAGTTTAGCTTCAATTCTAATTGATTGATTAATCAGTTGATTGGTTTCA<br>ATATGACAAATGGGTAGGGTGGGAAAACTTCATTTTCAATTCAGATCAAACTTTTTTGTTGTCGAC<br>ATAATATTTCTCGTTTGGGATGTTACTGTCACATTAATAATACACACACATCAGCTTATAATTTTG<br>AAAGTAATTTATCAGATATGTTGTGACGATCAATGGAAATGGCTAACTTCAATGTATCTGTTCTTC<br>CCCTTTTTCAAAGTTCACGTTTTTTGATTGATTGATTGATCTGTCGGCAGTGGTTTCAAAACCATTC<br>GGTGAGTAATCCTATCAATCAATGTTACGACAAAAGGCTCAATATTCAAAATTGCAATGTTTTAT<br>GTTTTCCTACGTGTACTTGTGCAAGGCAATTGATTCAACATTGCTTTTGGTGTTTGACGAGTTTCTA<br>GTTTGGACTTGTGTTGTTATCTGGGCTATACAGATTTCCCGGCTCACTATGAATTTTTTTTTTCGACG<br>CTCAGTGCACACAACTATAAACAACACAAACACAAACACAGCAAGAAAAAAAAAAAAACGAAC<br>ATTGAATTGAAACCAAGCCAACTGAAAAATTCCTTATTTAAATGACTGTCATACTAACCCATTTTT<br>ATAGAAGAAGTTGCTGCTTTAGTTATCGATAACGGTTCTCATATGAAAATTTCGGTGATCCCTGAG<br>CAGGTGGCGGAAACATTGGATGCTGAGAACCATTTCATTGTTCGTGAAGTGTTCGATGTGCACCT<br>ATCCGACCAAGGCTTTGAACTATCTACCAGAAGTGTGAGCCCCTACCGGAAGGATTACATCTCGG<br>ATGATGACTCTGATGAAGACTCTGCTTGCTATGGCGCATTCATCGACCAAGAGCTTGTCGGGAAG<br>ATTGAACTCAACTCAACATGGAACGATCTAGCCTCTATCGAACACATTGTTGTGTCGCACACGCA<br>CCGAGGCAAAGGAGTCGCGCACAGTCTCATCGAATTTGCGAAAAAGTGGGCACTAAGCAGACA<br>GCTCCTTGGCATACGATTAGAGACACAAACGAACAATGTACCTGCCTGCAATTTGTACGCAAAAT<br>GTGGCTTTACTCTCGGCGGCATTGACCTCTTCACGTATAAAACTAGACCTCAAGTCTCGAACGAA<br>ACAGCGATGTACTGGTACTGGTTCTCGGGAGCACAGGATGACGCCTAA
C. albicans URA3 terminator region	CATATGTGAAGTGTGAAGGGGGAGATTTTCACTTTATTAGATTTGTATATATGTATAATAAATAAA<br>TAAATAAGTTAAATAAATAATTAGATAAGGGTGGTAATTATTACTATTTACAATCAAAGGTGGTC<br>CTGCAT
